# Femtosecond Laser-Induced Graphene Modified with Platinum Nanoparticles for Advanced Multifunctional Sensing

**DOI:** 10.3390/s26134311

**Published:** 2026-07-07

**Authors:** Jie Zhan, Mingle Guan, Zi Wang, Xiaolin Qi, Sumei Wang

**Affiliations:** 1 Laser Micro/Nano Fabrication Laboratory, School of Mechanical Engineering, Beijing Institute of Technology, Beijing 100081, China; zhanjie0621@163.com (J.Z.); mingle0528@163.com (M.G.); 13966725970@163.com (Z.W.); qixl@bit.edu.cn (X.Q.); 2 Yangtze Delta Region Academy of Beijing Institute of Technology, Jiaxing 314000, China

**Keywords:** femtosecond laser, laser-induced graphene (LIG), multifunctional sensors, platinum nanoparticles (PtNPs)

## Abstract

Flexible sensors are important for wearable health monitoring, strain detection, and temperature sensing because of their mechanical flexibility and functional versatility. Here, a femtosecond laser direct scanning method was used to fabricate porous laser-induced graphene (LIG) and further modify it with platinum nanoparticles (PtNPs), forming Pt/LIG. This mask-free and rapid process enables simultaneous patterning and functionalization of flexible sensors. The introduction of PtNPs improves the electron transport and surface adsorption properties of LIG. As a result, the sheet resistance of Pt/LIG is reduced to 2.41 Ω/sq, enhancing electrical conductivity and suitability for sensing applications. Based on this method, highly sensitive strain and temperature sensors were fabricated. The Pt/LIG strain sensor shows a ΔR/R_0_ of 1141.8 at a bending angle of 90°, about 213% higher than that of pristine LIG, with fast response and recovery times of 36 and 56 ms, respectively. The temperature sensitivity also improved by about 650%, with a temperature coefficient of resistance of 0.240%/°C, compared with −0.032%/°C for pristine LIG. Overall, this work provides a fast and precise strategy for fabricating nanoparticle–graphene composites for flexible electronics, wearable health monitoring, and environmental sensing.

## 1. Introduction

The demand for flexible sensors in healthcare, human-machine interaction applications, and medical diagnostics is increasing because of their mechanical flexibility and stretchability, as well as the ability to conform to various surfaces [1,2,3]. A variety of materials such as advanced carbon materials, metallic nanomaterials, and conductive polymers have been extensively used in flexible and wearable electronic devices. Graphene has proven to be one of the most promising candidates among carbon nanomaterials due to its excellent physicochemical properties, including high electron mobility [4], good thermal stability and electrical conductivity [5], and high mechanical strength [6]. However, conventional graphene fabrication methods—including mechanical exfoliation [7,8], chemical vapor deposition (CVD) [9,10,11], epitaxial growth [12,13], and the reduction of graphene oxide [14,15]—are often limited by complicated procedures, potential chemical residues, the requirement for masks, and harsh processing conditions.

Therefore, laser direct scanning has become a feasible alternative for the facile fabrication of graphene on flexible polymer substrates [16,17,18,19]. Tour et al. demonstrated that the direct laser irradiation of polymer substrates lead to a three-dimensional porous structure of graphene, termed laser-induced graphene (LIG) [20]. LIG has become one of the most promising materials used in flexible sensors and electrodes. In addition to its high conformability and wearability, it can also detect various mechanical and chemical signals [21,22,23,24]. Recent examples include LIG on polyimide followed by the deposition of graphene oxide to form scalable multifunctional sensors [25], the femtosecond-laser conversion of Kevlar textiles into LIG for physiological strain monitoring [26], and the transfer of wood-derived LIG onto PDMS to form high-sensitivity thermistors [27], which collectively demonstrate that graphene fabricated by laser direct scanning can be applied in wearable electronics.

Beyond single-mode sensing, multimodal platforms have been realized by integrating LIG with complementary materials to broaden detection modalities. Examples include laser-induced porous carbon on starch-based films for simultaneous strain, temperature, and pressure detection [28], as well as paper-based graphene structures created via laser processing that enable biophysical and biochemical monitoring including strain, temperature, humidity, pH, and glucose sensing [29]. It has also been reported that a laser-induced sulfur-doped porous graphene (LISG) micro-supercapacitor was integrated with a separate carbon nanotube (CNT) foam-based pressure sensor for energy storage and humidity detection [30].

Metal nanoparticles are particularly effective functionalizers because they provide high electrical conductivity and catalytic activity that can enhance charge transport, interfacial chemistry and sensing transduction. The resulting hybrids exhibit uniformly distributed, firmly attached nanoparticles that increase defect density and provide catalytic/sensitization functionality, collectively enhancing charge transport, surface reactivity and device performance [31,32,33,34].

In this study, femtosecond laser processing is employed to fabricate laser-induced graphene (LIG) on flexible PEEK substrates and to further functionalize it with platinum nanoparticles (PtNPs). This method enables rapid, mask-free, transfer-free, and controllable fabrication of conductive porous graphene patterns on a flexible substrate. Compared with conventional laser-induced graphene sensors, the femtosecond laser process provides higher processing precision and lower thermal damage. By combining the porous conductive network of LIG with the high electrical conductivity and surface activity of PtNPs, the proposed Pt/LIG platform is expected to improve charge transport and sensing transduction. In addition, the incorporated PtNPs enhance the electrical conductivity and connectivity of the LIG network, which is beneficial for multifunctional sensing applications. This work focuses on the fabrication strategy, structural regulation, and sensing mechanism of PtNP-decorated LIG, aiming to provide a feasible route for developing high-performance flexible sensors for strain and temperature monitoring, wearable electronics, and human–machine interface applications. Future studies may further optimize metal species, nanoparticle distribution, and device integration to promote scalable and practical applications.

## 2. Materials and Methods

### 2.1. Material

A poly(ether-ether-ketone) (PEEK) film with a thickness of 150 μm was supplied by Junhua Special Polymer Materials Co., Ltd. (Junhua, Changzhou, China). Chloroplatinic acid (H_2_PtCl_6_·6H_2_O) was obtained from Beijing Mreda Technology Co., Ltd. (Beijing, China), and a solution with a concentration of 0.161 mol/L was prepared. Subsequently, 2 μL of the chloroplatinic acid solution was used for sensor fabrication.

### 2.2. Sensor Fabrication

**Figure 1:**
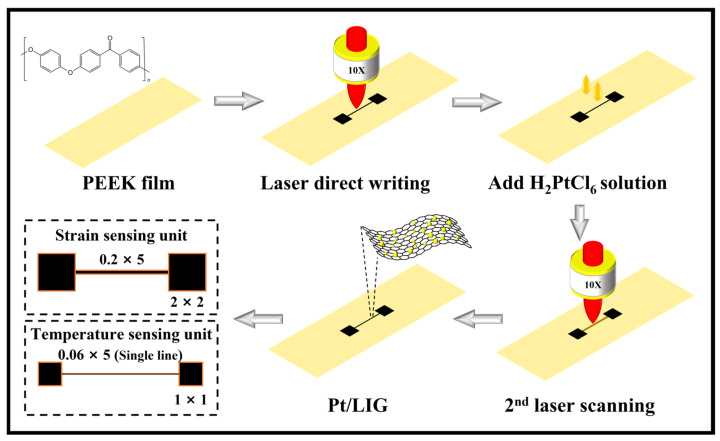
Schematic of fabrication steps of the Pt/LIG sensors. Unit of length: mm.

The Pt/LIG sensors were fabricated by a femtosecond laser direct-writing process, as illustrated in Figure 1. First, a 150 μm -thick PEEK film was cut into 1 cm × 3 cm pieces, cleaned with anhydrous ethanol, dried at 100 °C, fixed on a glass slide with adhesive tape, and mounted on a three-dimensional translation stage. A femtosecond laser system (YF-FL-050-200-IR) with a central wavelength of 1030 nm, pulse duration of 290 fs, and repetition rate of 1–800 kHz was used for laser processing. The laser beam was focused normally onto the front surface of the PEEK film through a 10× objective lens with a numerical aperture of 0.3. The scanning interval and scanning speed were set to 25 μm and 7 mm/s, respectively, and the laser power was 225 mW, corresponding to a laser fluence of 2.04 J/cm^2^ at a repetition rate of 800 kHz. According to the pre-designed sensing patterns, LIG was first fabricated by direct laser scanning, with sensing-unit dimensions of 5 mm in length and 0.2 mm and 0.06 mm in width for the strain and temperature sensors, respectively. Subsequently, 2 μL of 0.161 mol/L chloroplatinic acid solution was dropped onto the laser-processed area and dried. A second femtosecond laser scanning process was then performed on the sensing unit using the same laser fluence and scanning routine to promote the reduction, attachment, and anchoring of Pt nanoparticles onto the LIG surface. Finally, the sample was repeatedly rinsed with anhydrous ethanol and distilled water to remove unreacted chloroplatinic acid and then dried at room temperature. Copper wires were attached to the sensor electrodes using medical polyurethane tape, followed by the application of conductive silver paste, which was cured at 80 °C for 20 min in a vacuum drying oven. The sensor unit and electrodes were finally encapsulated with polyurethane tape to protect the device from external interference and mechanical damage during testing.

### 2.3. Characterization

The surface morphologies of the prepared LIG and Pt/LIG frameworks were characterized using a scanning electron microscope (SEM, S-4800, Hitachi, Tokyo, Japan). The Raman spectrum of the analyte was obtained using a high spectral resolution analytical grade micro-Raman imaging system with 532 nm laser excitation (inVia-Qontor). X-ray photoelectron spectroscopy (XPS) of LIG and Pt/LIG was conducted to investigate the chemical composition. XPS measurements were conducted using a PHI Quantera X-ray photoelectron spectrometer (ULVAC-PHI) with a monochromatic Al Kα (1486.6 eV) source. The lattice fringes and spacing of LIG and PtNPs were characterized by field emission gun transmission electron microscopy (TEM, TALOS F200X G2, Thermo Fisher, Waltham, MA, USA). The resistance variation of the sensor was recorded using a digital source meter under a constant low-current mode. A current of 1 mA was applied during the test.

Compression deformation tests were conducted using a DZPA100-ZJ23B motorized linear stage (20 μm resolution, ±3 μm repeatability) for strain sensing. Temperature response was evaluated with a DB-XGW far-infrared graphite heating plate under controlled thermal gradients.

## 3. Results and Discussions

### 3.1. Fabrication of the Pt/LIG Sensor

**Figure 2:**
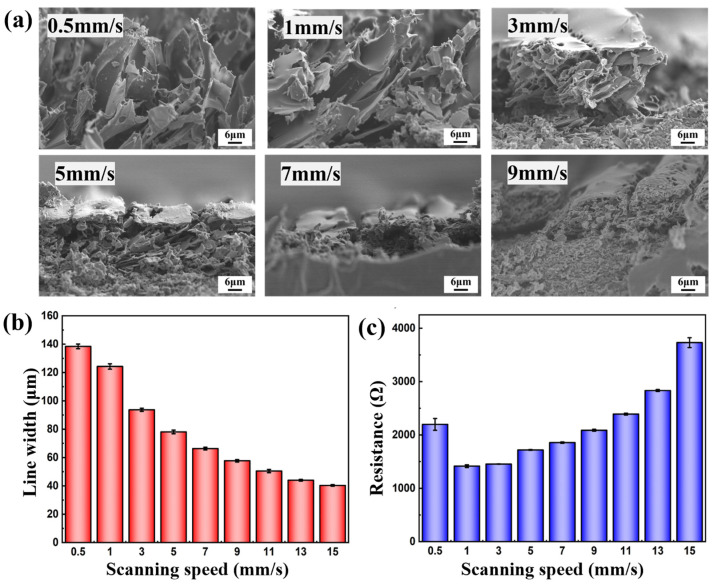
LIG single lines fabricated at different laser scanning speeds: (**a**) cross-sectional morphology, (**b**) line width, and (**c**) line resistance.

Figure 2a illustrates the cross-sectional morphology of laser-induced graphene (LIG) fabricated on PEEK. At scanning speeds below 3 mm/s, increased pulse energy deposition causes ablation and structural damage under repeated laser irradiation. As a result, a fibrous rib-like structure is formed. As the scanning speed increases (≥3 mm/s), the cross-section gradually evolves into a multilayer porous structure, and the depth of the laser-affected zone (LAZ) decreases significantly with further increases in scanning speed. At low scanning speeds, the prolonged laser–matter interaction leads to a deeper LAZ with a lot of pores of large sizes. At high scanning speeds, the laser irradiation time is shortened, the LAZ becomes shallower, and the pores tend to be denser with smaller sizes. The transient high temperatures induced by femtosecond laser irradiation generate photothermal and photochemical reactions, causing the C=O and C-O bonds to break and subsequently reorganize into new bonds, thereby forming porous graphene during the cooling process [35,36].

As shown in Figure 2b, the width of the laser-written lines decreases significantly from 138.4 μm to 40.4 μm as the scanning speed increases from 0.5 mm/s to 15 mm/s. This can be attributed to the reduced laser–material interaction at higher scanning speeds, which leads to lower energy deposition and effectively suppresses the LAZ. At low scanning speeds, heat accumulation induces more material melting and flow, resulting in a larger LAZ. In contrast, higher scanning speeds effectively limit thermal accumulation and diffusion.

Furthermore, as the scanning speed increases, the resistance of LIG first decreases and then increases, as shown in Figure 2c. At scanning speeds below the optimized value, ablation disrupts the conductive pathways and increases resistance. Conversely, when the scanning speed is above the optimized value, insufficient energy deposition leads to incomplete carbonization and graphitization, which also increases resistance. Therefore, an appropriate scanning speed of 7 mm/s is essential to maintain structural integrity and achieve a high graphitization degree, enabling the fabrication of LIG with small resistance and providing a suitable space for the subsequent adhesion of platinum nanoparticles (PtNPs).

**Figure 3:**
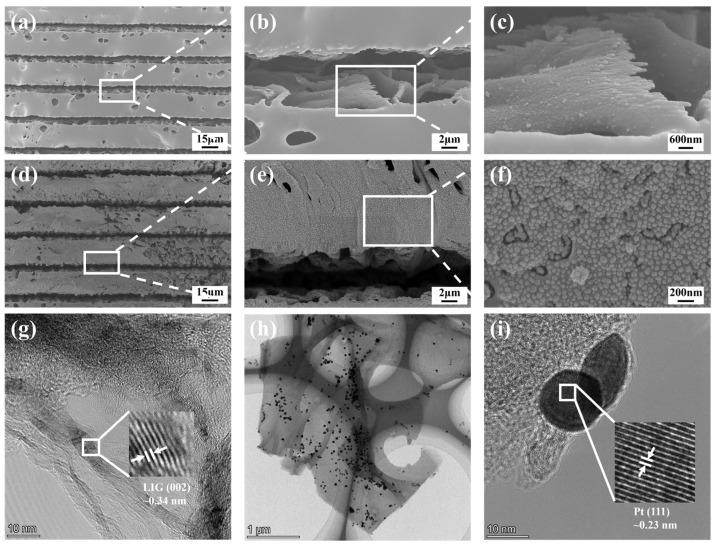
Structural characterization of LIG and Pt/LIG: SEM images of (**a**–**c**) LIG and (**d**–**f**) Pt/LIG, and TEM images of (**g**) LIG and (**h**,**i**) Pt/LIG. The laser fluence is 2.04 J/cm^2^.

Figure 3 shows the SEM and TEM results used to characterize the structure of LIG and Pt/LIG. As shown in Figure 3a–c, a wrinkled network morphology of LIG is observed within the LAZ, with porous regions between adjacent LAZs. Figure 3d–f reveal that after a second laser irradiation, the LIG surface is uniformly decorated with densely distributed nanoparticles. The resistance values measured under various processing conditions are summarized in Table S1. Compared with directly double femtosecond laser scanning, treatment with chloroplatinic acid solution reduces the sample resistance from 22.7 to 2.41 Ω/sq. The contact between PtNPs and graphene induces charge transfer, increasing carrier concentration in LIG. Uniformly distributed PtNPs act as highly conductive bridges between graphene sheets, enhancing the connectivity of the porous LIG network. PtNPs can also enhance local graphitization during the second laser scanning, thereby suppressing carrier scattering and promoting more efficient charge transport.

TEM images of the wrinkled graphene sheets are displayed in Figure 3g, with a lattice spacing of approximately 0.34 nm, consistent with the (002) plane of graphene [37]. Figure 3h,i further demonstrate evenly distributed nanoparticles on the graphene surface, with a measured lattice spacing of 0.23 nm corresponding to the (111) crystallographic plane of face-centered cubic (fcc) platinum [38]. These observations confirm the successful synthesis and incorporation of crystalline PtNPs within the LIG.

### 3.2. Characteristics of the Pt/LIG

**Figure 4:**
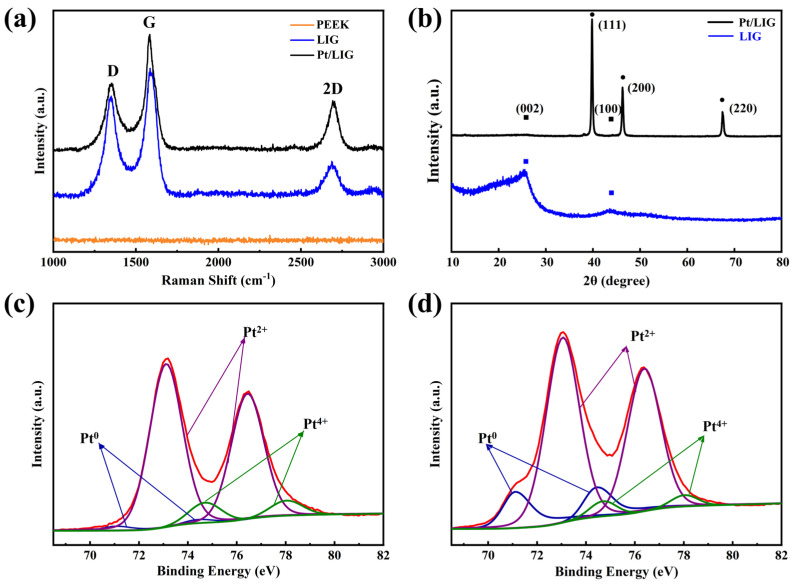
(**a**) Raman spectra of PEEK, LIG, and Pt/LIG. (**b**) XRD patterns of Pt/LIG and LIG. XPS spectra of (**c**) LIG with added chloroplatinic acid and (**d**) Pt/LIG.

Raman spectroscopy, XRD and XPS were used to characterize the Pt/LIG samples. As shown in Figure 4a, the Raman spectrum of LIG exhibits three characteristic peaks: the D peak at ~1354 cm^−1^, which is commonly attributed to defects and lattice disorganization of graphene or bending of sp^2^ carbon bonds; the G peak at ~1584 cm^−1^ that is associated with the in-plane vibrational modes of sp^2^-bonded carbon atoms; and the 2D peak at ~2693 cm^−1^, associated with second-order phonon processes concerning the graphene lattice structure at the graphite boundary. The intensity ratios of these peaks, particularly the I_D_/I_G_ and I_2D_/I_G_ ratios, serve as critical indicators of the quality and structural features of the graphene in the LIG. A significant decrease in the I_D_/I_G_ ratio for Pt/LIG samples, from 0.78 to 0.58, indicate a notable reduction in defect density within the graphene. At the same time, the I_2D_/I_G_ ratio increased markedly from 0.27 to 0.42, indicating that the number of graphene layers decreased and the overall crystallinity also improved. The reduction in defect density within the graphene is manifested by the declining I_D_/I_G_ ratio, whereas a decrease in the number of graphene layers is indicated by the increasing I_2D_/I_G_ ratio [39]. These spectral changes collectively demonstrate that the second laser scanning process effectively promotes further graphitization of the LIG.

As shown in Figure 4b, the pristine PEEK substrate exhibited diffraction peaks at 2θ = 18.96°, 20.72°, 22.34°, and 28.65°, which correspond to the (110), (111), (200), and (211) crystal planes, respectively [40]. After laser direct writing, the diffraction pattern of the LIG sample changed markedly, with a prominent peak emerging at 2θ ≈ 26°, which corresponded to the (002) phase of graphene. This finding is consistent with the interlayer spacing and crystal planes observed in the TEM images (Figure 3g). Additional distinct diffraction peaks appeared at 2θ values of approximately 39.79°, 46.28°, and 67.53°, which corresponded to the (111), (200), and (220) crystal planes of fcc platinum, respectively [38]. These peaks also confirm the successful incorporation and crystallization of PtNPs on the LIG network. The presence of these well-defined platinum diffraction peaks not only affect the effective deposition of platinum but also its crystalline properties.

The Pt 4f XPS spectrum before second laser scanning is presented in Figure 4c and evidently shows that platinum occurs predominantly as Pt^2+^ and Pt^4+^, attributable to the chloroplatinic acid precursor used in preparation. At this stage, the spectrum shows only a negligible contribution from metallic platinum (Pt^0^). After the second laser scanning, changes are noted in the Pt 4f XPS spectrum as shown in Figure 4d, where there is a significant increase in the intensity of peaks attributed to metallic Pt^0^ [41], suggesting effective laser-induced reduction of the platinum species. Chemical state component changes of Pt before and after laser treatment are summarized in Table S2. Specific Pt peaks at lower binding energies of approximately 71.1 eV (Pt 4f7/2) and 74.4 eV (Pt 4f5/2) are introduced through the laser reduction process, resulting in a change in the shape of the superposed peak. These findings indicate that second laser scanning is an effective way to convert oxidized platinum species to the metallic state, confirming the reduction mechanism facilitated by femtosecond laser scanning.

In addition to the Pt 4f signals, the evolution of the C- and O-related XPS peaks provides further evidence for the chemical transformation induced by the reported processing. For pristine PEEK, the C 1s spectrum contains contributions from aromatic carbon and oxygen-containing carbon species, including C-O and C=O groups originating from the ether and ketone units in the polymer backbone. After femtosecond laser irradiation, the C 1s envelope of LIG is mainly dominated by the sp2-hybridized C=C/C-C component at approximately 284.5 eV, whereas the relative intensities of oxygen-containing components such as C-O, C=O, and O-C=O are reduced. Correspondingly, the O 1s signal becomes weaker after laser conversion, indicating partial removal of oxygen-containing functional groups during the photothermal carbonization of PEEK. Such behavior is consistent with previous XPS studies on graphene-derived materials, in which thermal or laser-assisted reduction lead to deoxygenation and restoration of the conjugated carbon network [42].

For Pt/LIG, the C 1s spectrum still reflects the graphitized carbon framework of LIG, suggesting that the introduction of chloroplatinic acid and the subsequent second laser scanning do not destroy the conductive carbon network. The residual O 1s contribution in Pt/LIG can be assigned to the remaining oxygen-containing surface groups, adsorbed oxygen/water, and possible interfacial Pt-O or Pt-O-C species formed during the laser-assisted reduction of the platinum precursor. Therefore, the changes in both the C 1s and O 1s spectra demonstrate that the processing not only promotes the formation of conductive LIG and metallic Pt nanoparticles but also modifies the surface oxygen chemistry, which may facilitate PtNP anchoring and contribute to the sensing performance of the Pt/LIG device.

### 3.3. Sensor Performance Test and Application

#### 3.3.1. Strain-Sensing Performance

The strain-sensing performance of LIG was first tested, the relative resistance change (ΔR/R_0_) increased continuously with increasing bending angle. At large bending angles, the conductive pathways are easily damaged, resulting in significant resistance variations. At small bending angles, the conductive pathways are relatively stable, and the resistance changes slightly. Therefore, the experimental setup shown in Figure S3a was used to preprocess LIG. The surface morphologies of LIG with different compression distances are shown in Figure S3b–f. To ensure the durability of the Pt/LIG sensor, a compression distance of 5 mm was used, and after two compression cycles, chloroplatinic acid solution was added and the sample was subjected to a second laser scanning.

**Figure 5:**
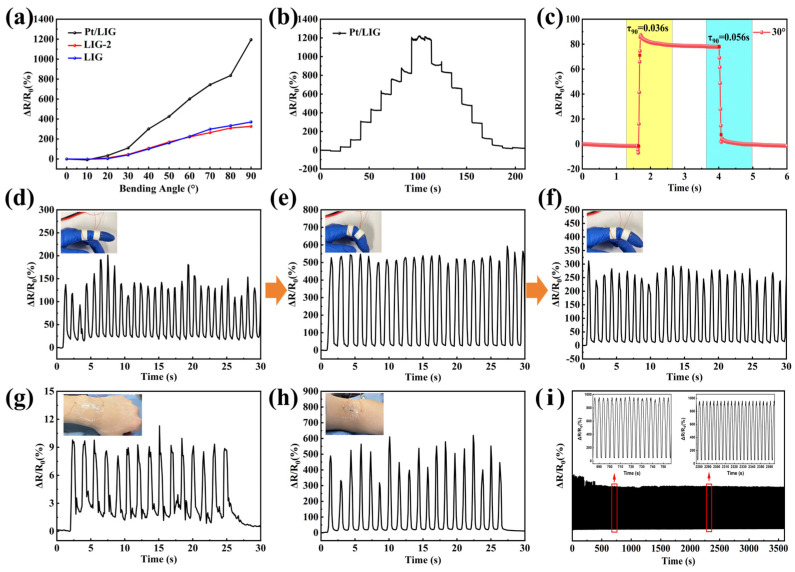
(**a**) Variations in ΔR/R_0_ as a function of bending angle with LIG, LIG-2, and Pt/LIG sensors. Pt/LIG strain sensor performance: (**b**) ΔR/R_0_ measured during the bending and releasing process from 0 to 90°. (**c**) Response and recovery times of the sensor. Real-time motion detection using the Pt/LIG strain sensor. (**d**–**f**) Sensor response to progressively increasing finger flexion angles (index finger mounting). (**g**) Wrist-mounted sensor demonstrating high sensitivity to subtle movements. (**h**) Elbow-mounted sensor monitoring large-amplitude arm motions. Insets: Corresponding images of the sensor during testing (Clear insets are shown in Figure S4). (**i**) Stability observed during a one-hour cycling test. The ΔR/R_0_ values were all measured at room temperature (25 °C).

To investigate the strain-sensing performance of Pt/LIG, ΔR/R_0_ was measured during bending and release, with the bending angle increased from 0° to 90° in 10° increments and maintained for 10 s at each angle. As shown in Figure 5a, the ΔR/R_0_ values of LIG, LIG-2, and Pt/LIG increase with increasing bending angle, which is attributed to the bending-induced opening of microcracks and the reduced number of conductive pathways in the porous LIG network. At 90°, the ΔR/R_0_ values of Pt/LIG, LIG, and LIG-2 are 1141.8, 365.2, and 347.0, respectively. Thus, Pt/LIG shows a 213% increase compared with LIG and a 229% increase compared with LIG-2. This improvement is mainly attributed to the incorporation of PtNPs, which decrease the initial resistance R_0_ by forming conductive bridges between graphene sheets. Meanwhile, the PtNP-assisted junctions are sensitive to crack opening and contact resistance variation during bending, resulting in a larger normalized resistance change.

As shown in Figure 5b, the resistance response of Pt/LIG during bending and release from 0° to 90° follows similar variation trends, indicating reversible reconstruction of the conductive network. During bending, the separation between adjacent conductive domains increases, leading to increased resistance; during release, partial crack closure restores conductive contacts and reduces resistance. Figure 5c shows the transient response of the Pt/LIG sensor, with response and recovery times of 36 and 56 ms, respectively. The rapid response arises from direct resistance modulation of the porous Pt/LIG network, without slow diffusion- or adsorption-controlled processes.

The Pt/LIG strain sensor shows strong potential for human motion monitoring due to its excellent sensing performance. As the finger-bending angle increases, ΔR/R_0_ correspondingly rises. At similar bending angles, the sensor exhibits accurate and repeatable responses, as illustrated in Figure 5d–f. The sensor exhibits extremely high sensitivity in wrist-motion monitoring, precisely capturing changes in resistance and providing real-time feedback (Figure 5g). During larger arm movements, the sensor also responds rapidly and generates well-defined resistance-change signals (Figure 5h). In summary, these results indicate that the sensor can reliably resolve both small- and large-amplitude movements and produce signals with a high signal-to-noise ratio suitable for real-time monitoring. Also, a bending-release cycling test was also conducted for 1 h, and the sensor maintained a steady response over the measurement period, as depicted in Figure 5i.

#### 3.3.2. Temperature-Sensing Performance

To investigate the temperature-sensing performance of the LIG sensor, resistances were measured as the temperature increased at 10 °C increments, with each temperature step maintained for 3 min to evaluate sensitivity. The temperature coefficient of resistance (*TCR*) was calculated using Equation (1), which characterized the sensitivity of the temperature sensor [43,44]:

(1) $$TCR=\frac{\left(R-R_{0}\right)/R_{0}}{T-T_{0}}$$

In Equation (1), *R*_0_ is the resistance of the sensor at the initial temperature *T*_0_, and *R* is the resistance corresponding to the higher temperature *T*.

**Figure 6:**
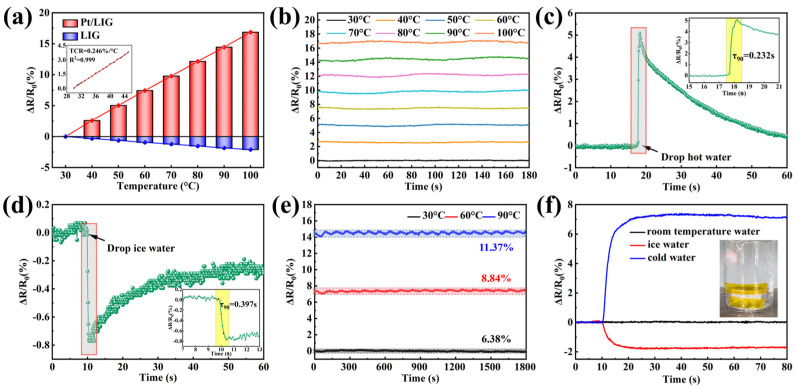
(**a**) *ΔR*/*R*_0_ as a function of temperature from 30 to 100 °C with Pt/LIG and LIG sensors, the inset shows *ΔR*/*R*_0_ from 30 to 45 °C. Pt/LIG temperature-sensing performance: (**b**) resistance measurements from 30 to 100 °C taken every 10 °C for 3 min. Sensor response to thermal shock induced by (**c**) a drop of hot water and (**d**) a drop of ice water. (**e**) Measurements conducted for 30 min at 30 °C, 60 °C, and 90 °C. (**f**) Detection of various real-time signals by the Pt/LIG temperature sensor fixed on the outer side of a beaker with the addition of cold water, hot water, and room temperature water, respectively. The inset show actual images of the sensor during testing.

Within the temperature range of 30 to 100 °C, a comparison of the temperature-sensing performance between LIG and Pt/LIG is presented in Figure 6a. In contrast to the LIG sensor, whose resistance decreases with increasing temperature, the Pt/LIG sensor exhibits a positive *TCR*, with resistance increasing as temperature rises. This behavior is consistent with that of platinum, which has a positive *TCR* of approximately 0.39%/°C [45]. And resolution is critical for practical temperature-sensing applications, so the inset shows that the Pt/LIG sensor can achieve a temperature resolution of 1 °C within the 30–45 °C range, making it highly suitable for human body temperature detection. As illustrated in Figure S6a, the |*TCR*| of the Pt/LIG temperature sensor is significantly higher than that of LIG and LIG-2, reaching 0.240%/°C, which is about 650% higher than that of LIG (−0.032%/°C). In contrast, LIG-2 shows no significant change in sensitivity, indicating that the enhancement in sensing performance is closely related to PtNPs doping. The positive *TCR* of the Pt/LIG temperature sensor is attributed to the metallic properties introduced by the PtNPs, in which charge carrier scattering governs the temperature dependence of its resistance. As the temperature rises, increased phonon scattering of charge carriers reduces the mean free path and consequently lowers the mobility, which leads to an increase in resistance [46,47].

Figure 6b shows the resistance variations of the sensor during temperature testing, demonstrating its high stability and repeatability. Each temperature test was repeated three times. Figure S6b shows that the ΔR/R_0_ at each temperature are nearly identical, further demonstrating the sensor’s excellent repeatability. In addition, the response of the sensor to temperature fluctuations was evaluated by applying drops of hot water and ice water on the sensor surface. The results indicate that the sensor responds to sudden temperature increases and decreases with response times of 0.232 s (Figure 6c) and 0.397 s (Figure 6d), respectively. To evaluate long-term stability, the sensor was continuously monitored for 30 min at each temperature (30 °C, 60 °C and 90 °C). As shown in Figure 6e, the resistance remained stable during each measurement interval. The recorded fluctuations in *ΔR*/*R*_0_ were 6.38%, 8.84%, and 11.37% at 30 °C, 60 °C, and 90 °C, respectively. These moderate changes can be attributed to small variations in the heating stage and ambient temperature during the testing period. Overall, the results indicate good thermal stability of the sensor under various temperature conditions.

As shown in Table 1, the temperature-sensing performance of Pt/LIG sensors fabricated by a femtosecond laser is comparable to or better than that of other reported single-function temperature sensors. The Pt/LIG temperature sensor exhibits a sensitivity of 0.240%/°C, a response time of 0.232 s and a recovery time of 0.397 s, indicating strong potential for integration into multifunctional wearable devices for health management.

During dynamic temperature monitoring, the sensor was attached to the outside of a beaker, and equal volumes of cold, hot, and room temperature water were added sequentially. As illustrated in Figure 6f, the sensor accurately detects changes in water temperature, demonstrating excellent dynamic response and external temperature-sensing capability. In summary, the Pt/LIG temperature sensor is an accurate, flexible wearable device that could meet various temperature monitoring needs.

## 4. Conclusions

This study presents a femtosecond laser double-scanning strategy for fabricating Pt/LIG flexible multifunctional sensors, in which porous LIG is first directly generated on a PEEK film and Pt nanoparticles are then reduced and anchored onto the LIG network during the second laser scanning, integrating LIG patterning and PtNP functionalization into a laser-based process. This method avoids complex masks and multistep chemical treatments, enables localized processing on flexible substrates, and provides a simplified route for preparing metal-nanoparticle/LIG composites. The incorporation of PtNPs improves the conductive connections and local graphitization of the porous LIG network, reducing the sheet resistance of Pt/LIG to 2.41 Ω/sq; Raman, XRD, TEM, and XPS results confirm the formation of PtNPs on LIG and the laser-induced reduction of Pt species. For strain sensing, the Pt/LIG sensor exhibits a ΔR/R0 of 1141.8 under 90° bending, which is approximately 213% greater than that of the pristine LIG sensor, with response and recovery times of 36 ms and 56 ms, respectively. It also distinguishes motion signals from finger, wrist, and elbow movements and maintains stable responses during a 1 h bending-release cycling test. For temperature sensing, the Pt/LIG sensor shows a positive temperature coefficient of resistance over 30–100 °C, with a *TCR* of 0.240%/°C, approximately 650% greater in magnitude than that of pristine LIG (−0.032%/°C); resolves temperature changes of 1 °C; and exhibits response and recovery times of 0.232 s and 0.397 s, respectively, while also detecting dynamic water-temperature changes. Benefiting from the flexibility of the PEEK substrate and the sheet resistance of the Pt/LIG network of 2.41 Ω/sq support the application of the sensor in wearable human motion monitoring, pressure detection, temperature sensing, and integrated flexible electronic systems. This work demonstrates the advantages of femtosecond-laser-fabricated Pt/LIG sensors for strain and temperature detection and provides a feasible platform for flexible electronics, wearable health monitoring, and integrated multifunctional sensing systems.

## Figures and Tables

**Table 1:** Performance comparison of temperature sensor.

Material	Detecting Range	Sensitivity	Response/Recovery Time	Ref.
LIG	30–80 °C	0.089%/°C	-	[48]
Cu/LIG	25–140 °C	0.055%/°C	0.142 s/0.147 s	[34]
NiO	24–40 °C	3.84%/°C	0.37 s/0.56 s	[44]
Gr/PEO/PVDF	25–42 °C	-	26 s/-	[49]
GNWs/PDMS	25–120 °C	0.214%/°C	1.6 s/8.52 s	[50]
Ag/PI	20–60 °C	0.223%/°C	-	[51]
pNIPAM/ PEDOT:PSS/ CNT	25–40 °C	2.6%/°C	167 s/605 s	[52]
PEDOT:PSS/ CNTs/PET	30–55 °C	0.85%/°C	-	[53]
Graphene	−20–100 °C	0.12%/°C	1 s/3.3 s	[54]
Pt/LIG & GO	30–100 °C	0.240%/°C	0.232 s/0.397 s	This work

## Data Availability

Data available on request from the authors.

## Supplementary Materials

The following supporting information can be downloaded at: https://www.mdpi.com/article/10.3390/s26134311/s1.
