# Supplementary material for: Femtosecond Laser-Induced Graphene Modified with Platinum Nanoparticles for Advanced Multifunctional Sensing
Source: Sensors (Basel). 2026 Jul 7;26(13):4311. doi: 10.3390/s26134311 (PMC13364155; doi:10.3390/s26134311)
Supplement: Supplementary file 1 [file sensors-26-04311-s001.zip › sensors-4315014-supplementary.pdf]

Supporting Information

Femtosecond Laser-Induced Graphene Modified with Platinum Nanoparticles for Advanced Multifunctional Sensing

Jie Zhan[a], Mingle Guan[a,b], Zi Wang[a], Xiaolin Qi[a], Sumei Wang[a,b,*]

[a] *Laser Micro/Nano Fabrication Laboratory, School of Mechanical Engineering, Beijing Institute of Technology, Beijing 100081, China*

[b] *Yangtze Delta Region Academy of Beijing Institute of Technology, Jiaxing 314000, China*

* *E-mail: wangsumei@bit.edu.cn*

Sensor Preparation:

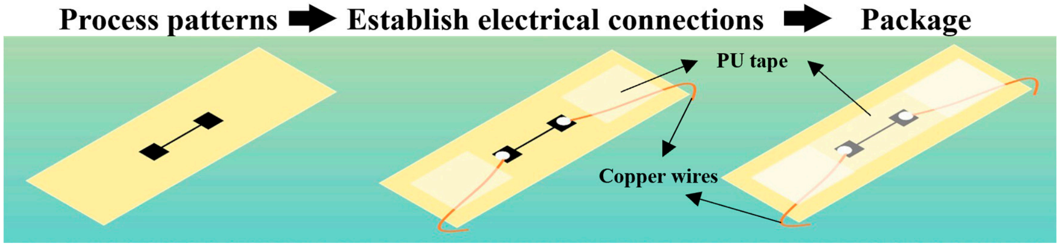

Figure S1. Schematic illustration of the fabrication process for both strain and temperature sensors.

Characteristics of the Pt/LIG:

Table S1. Resistance values measured under various processing conditions. The insets show the measurement method of resistance.

**Processing conditions** 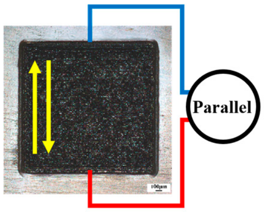	**Resistance values/Ω**
Scan at 7 mm/s	23.866±0.710
Scan twice at 7 mm/s	22.719±1.057
Scan at 7 mm/s→Add chloroplatinic acid solution and dry→Scan at 7 mm/s→Rinse and dry	**2.412±0.341**

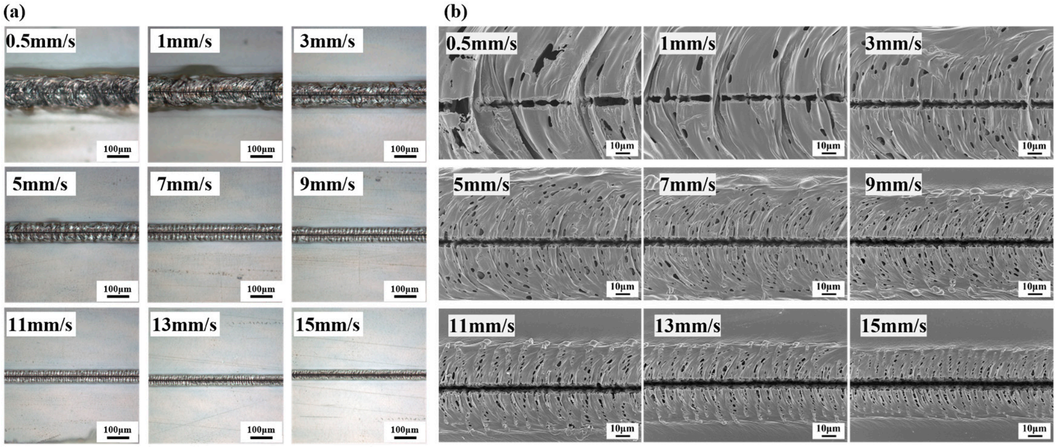

Figure S2. LIG single lines fabricated at different laser scanning speeds: (a) OM images and (b) SEM images.

Table S2. XPS chemical state components of Pt in samples after drying the chloroplatinic acid solution, measured before and after second laser scanning.

Sample	Pt^0 $4f_{7/2}$	Pt^{2+} $4f_{7/2}$	Pt^0 $4f_{5/2}$	Pt^{4+} $4f_{7/2}$	Pt^{2+} $4f_{5/2}$	Pt^{4+} $4f_{5/2}$
	71.1±0.1 eV	73.1±0.1 eV	74.4±0.1 eV	74.7±0.1 eV	76.4±0.1 eV	78.0±0.1 eV
Before second laser scanning	1.39%	50.1%	1.04%	5.65%	37.58%	4.24%
After second laser scanning	**8.96% ↑**	45.42%	**6.72% ↑**	2.76%	34.07%	2.07%

Strain sensor:

The motorized translation stage was operated at a speed of 35 mm/s with an acceleration of 70 mm/s². The sensor was compressed by 2.5, 5.0, 7.5, and 10.0 mm, each repeated twice, using the test setup shown in Figure S4(a). SEM images (Figure S4(b–f)) revealed no significant cracks in the sensor under 2.5 mm and 5 mm compression. At 7.5 mm compression, cracks appeared on the LIG surface. At 10 mm compression, severe cracks caused irreversible damage to the sensor.

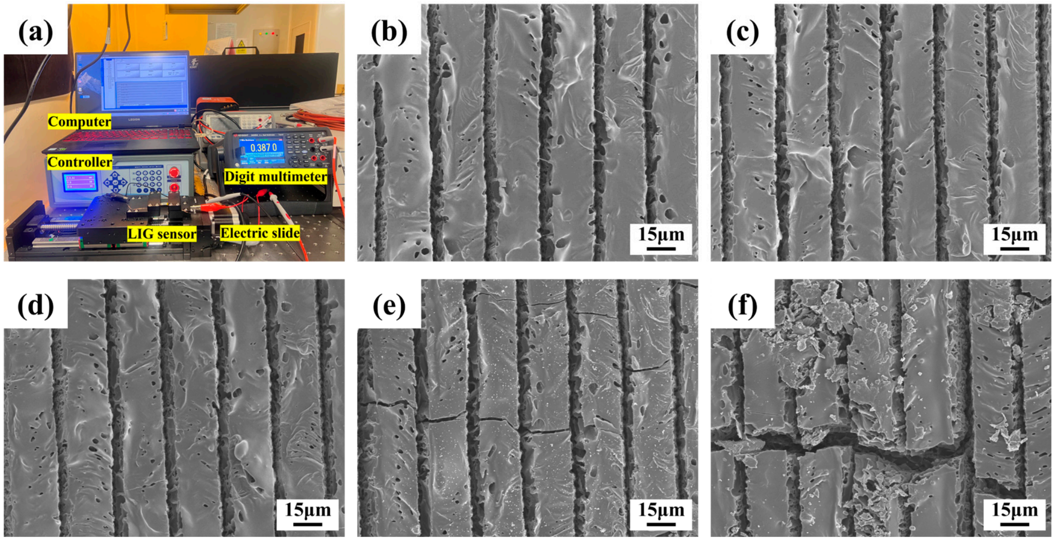

Figure S3. (a) Photographs of the test platform. SEM images of the LIG surface under varying compression distances: (b) 0 mm, (c) 2.5 mm, (d) 5.0 mm, (e) 7.5 mm, and (f) 10 mm.

Figure S3 presents the resistance change rates of the Pt/LIG, LIG-2 (subjected to a second laser scan without chloroplatinic acid), and LIG sensors after two cycles of 5 mm compression followed by a bending angle of 90°.

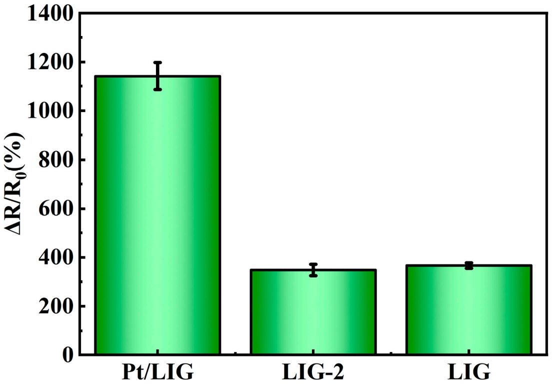

Figure S4. Resistance change rate at a bending angle of 90°.

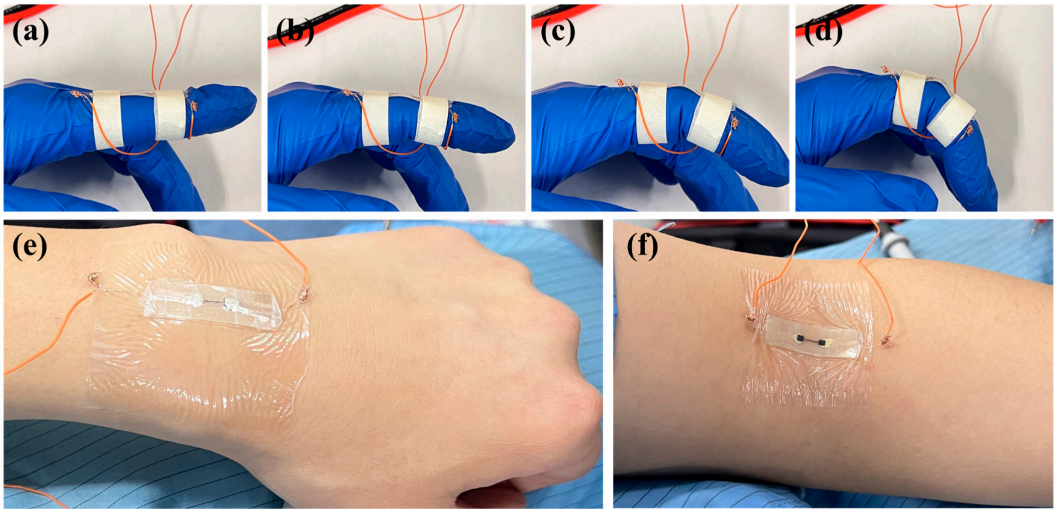

Figure S5. Bending stress tests conducted on Pt/LIG sensors attached to (a-d) finger joints, (e) the wrist and (f) the elbow.

Temperature sensor:

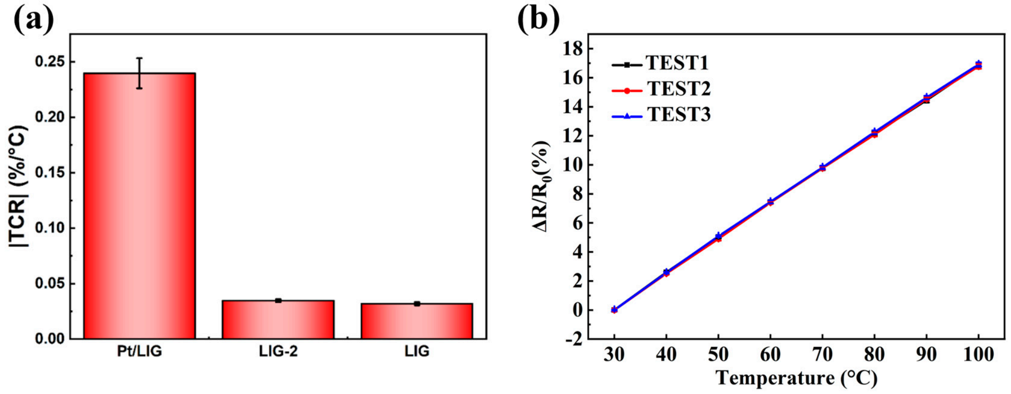

Figure S6. (a) Temperature-sensing sensitivity of Pt/LIG, LIG-2, and LIG based on resistance changes measured from 30 to 100 °C. (b) $\Delta R/R_0$ of the sensor under gradient temperature changes from 30 °C to 100 °C during three test cycles.
